# Comparison of the efficacy and perioperative pain between vessel sealing and suture ligation for median celiotomy in canine ovariohysterectomy

**DOI:** 10.14202/vetworld.2023.386-394

**Published:** 2023-02-28

**Authors:** Jutapoln Sunghan, Sareepah Manmoo, Wanna Suriyasathaporn, Witaya Suriyasathaporn, Kanawee Warrit, Pradipa Kusolphat

**Affiliations:** 1 Faculty of Veterinary Science, Prince of Songkla University, Hat Yai, 90110, Thailand; 2 Department of Companion Animal and Wildlife Clinic, Faculty of Veterinary Medicine, Chiang Mai University, Chiang Mai 50100, Thailand; 3 Research Center of Producing and Development of Products and Innovations for Animal Health and Production, Chiang Mai University, Chiang Mai 50100, Thailand; 4 Cambodia Campus, Asian Satellite Campuses Institute, Nagoya University, Nagoya, Japan

**Keywords:** ligation, ovariohysterectomy, post-operative pain, vessel sealing

## Abstract

**Background and Aim:**

Vessel sealing (VS) is used widely in human medicine and veterinary practice during laparoscopic surgery; however, few studies have investigated VS in canine ovariohysterectomy (OHE) using the median celiotomy approach. This study aimed to compare the effect of VS and suture ligation (SL) on surgical time, blood loss, and perioperative pain in canine OHE through median celiotomy.

**Materials and Methods:**

Twenty-eight dogs were randomly and equally assigned into two groups that underwent surgery either by SL at both the ovarian pedicle and uterus or using a disposable VS device. The short form of the Glasgow composite pain scale (SF-GCPS) and the Colorado state university canine acute pain scale (CSU-CAP) were used to determine pain pre-operatively (baseline); at 30 min; and at 1, 2, 3, 4, 24, and 72 h post-operatively. Perioperative physiological parameters, surgical duration, and percentage of blood loss were recorded. Repeated measures analysis was performed to determine the differences in all parameters among time-related tasks and between both groups. A significant difference was defined at p < 0.05.

**Results:**

The duration from identification of the first ovary to uterus removal was shorter in VS than in SL (p < 0.05). No clinically relevant differences were found among physiological variables. Both groups showed higher SF-GCPS and CSU-CAP values after surgery compared with baseline. The SF-GCPS in SL at 1 h was higher than in VS (p < 0.05). Two dogs in the SL group required additional post-operative rescue analgesia. No differences were found between the groups in terms of blood loss.

**Conclusion:**

The use of a VS device in dogs undergoing OHE celiotomy decreased post-operative pain and shortened the perioperative time, making it an effective alternative technique for this common surgery. However, the VS device must be applied 2–3 times in the same location during the OHE procedure to prevent technical failure. This disposable device was reused up to 5 times for economic reasons without device failure. Soft tissue damage during OHE using the VS device should be investigated in a future prospective study.

## Introduction

Ovariohysterectomy (OHE) is one of the most common surgical procedures on the female reproductive system in veterinary practice [1]. Ovariohysterectomy is performed to remove the ovaries, uterine horns, and uterine body [2, 3]. This procedure is a safe and efficient method for controlling the animal population and decreasing the risk of life-threatening diseases, such as pyometra [4] and mammary gland tumors [5]. Ovariohysterectomy also reduces the erratic behaviors associated with the estrous cycle [1].

Noxious pain stimuli during the surgical procedure, including stretching, inflammation, and ischemia, can cause visceral pain [6, 7]. In addition, ineffective post-operative pain control increases the risk of mortality [8], development of postsurgical complications [9], and chronic pain, the management of which is more difficult [10]. The use of suture ligation (SL) in OHE also causes perioperative pain [11–14], morbidity from tissue trauma [15], organ manipulation [15], inflammation [16], and hemorrhage from the left and right ovarian arteries [17]. Thus, adequate hemostatic techniques are essential during and after surgery for pain reduction [5, 18] and the reduction of hemorrhage-related morbidity [19, 20].

At present, vessel sealing (VS) devices are widely used in human and veterinary medicine for vessel coagulation and incision [21–23]. Such devices fuse the vessel walls and create seals using a combination of electrical current and mechanical pressure [24], resulting in significantly shortened surgery time [25], excellent hemostasis [19], and less pain and surgical stress in OHE [20]. Although the VS approach is not necessarily novel, the assessment of perioperative pain and determining the amount of tissue damage expected of the technique has rarely been investigated in small animal surgery practice [12, 15, 26]. Furthermore, studies comparing post-operative pain after midline celiotomy OHE through a bipolar VS device and SL are limited.

Therefore, this study compared the surgical time, percentage of blood loss, perioperative pain, and the physiological factors related to pain between the VS and SL methods of median celiotomy in canine OHE. Findings should be valuable to veterinary surgeons in providing data-based decisions in the selection of either VS or ligature for OHE.

## Materials and Methods

### Ethical approval

The research protocol was approved by the Institutional Animal Care and Use Committee, Prince of Songkla University (2563-05-054). The owner’s consent was obtained before using their dog for the study.

### Study period and location

The study was conducted from October 2020 to October 2021 at the Small Animal Hospital, Faculty of Veterinary Science, Prince of Songkla University, Thailand.

### Animals

This study was performed as a prospective randomized clinical trial. Twenty-eight healthy female dogs ranging from 1 to 5 years of age and of any breed selected for OHE were assigned by simple randomization into the SL or VS group (n = 14 in each group). All female dogs were healthy and American Society of Anesthesiologists category I or II. Physical examination, complete blood count, and serum chemistry profile (alanine aminotransferase, alkaline phosphatase, blood urea nitrogen, and creatinine) were performed pre-operatively. The parameters for all dogs were within the normal reference ranges. The exclusion criteria used were aggressiveness, fear, signs of pre-existing pain or inflammation, underlying diseases, and continued anti-inflammatory, corticosteroid, or analgesic drugs within 7 days before the study.

### Anesthetic protocol

Before anesthesia, water and food restrictions were imposed at 4–6 h and 8 h pre-operatively, respectively. All dogs were pre-anesthetized with diazepam (0.3 mg/kg, intravenously [IV], diazepam; The Government Pharmaceutical Organization, Thailand) and tramadol hydrochloride (3 mg/kg, IV, tramadol analgesic; TP. Drug Laboratories Co. Ltd., Thailand). Subsequently, general anesthesia was induced with propofol (4–6 mg/kg, IV, POFOL; Dongkook Pharmaceutical, Korea) and maintained with isoflurane (1%, Aerrane isoflurane USP; Baxter Healthcare Corporation, USA) and 100% oxygen (40 mL/kg/h) via endotracheal tube intubation in a circle rebreathing system. Cefazolin (25 mg/kg, IV, Cefazillin^®^; TP. Drug Laboratories Co. Ltd.) was administered at the time of induction as prophylaxis. Lactated Ringer’s solution (Lactated Ringer’s injection, General Hospital Products Public Co., Ltd., Thailand) was infused IV at 10 mL/kg/h during anesthesia. A suitable surgical anesthesia plane (a ventral eye position, relaxed jaw tone, and palpebral reflex absence) was maintained by adjusting the isoflurane vaporizer setting [15]. Lidocaine (2 mg/kg, lidocaine hydrochloride; The Government Pharmaceutical Organization, Thailand) and bupivacaine (0.5 mg/kg, Marcain^®^ 0.5%, AstraZeneca [Thailand] Ltd., Thailand) were infiltrated into the surgical site before incision.

Continuous electrocardiography, heart rate, respiratory rate, body temperature, end-tidal CO_2_, isoflurane, pulse oximetry SpO_2 (_(iPM 12 Vet, Mindray, Shenzhen Mindray Bio-Medical Electronics Co. Ltd., China), and indirect systolic blood pressure (using a Doppler ultrasonic sphygmomanometer, [vmed Vet-Dop2™, Vmed Technology, Washington, United States]) were monitored and recorded every 5 min during anesthesia. All dogs received a circulating water blanket (Soarmed Medical-Tech Co., Ltd, Taiwan) to prevent hypothermia. The tidal volume was 10 mL/kg. The ventilation was controlled to maintain end-tidal CO_2_ levels between 35 and 45 mmHg throughout anesthesia. The dogs in both groups were injected with carprofen (4.4 mg/kg, subcutaneously, Rimadyl; Zoletil^®^, Brazil) immediately after extubation for the relief of post-operative pain and inflammation.

### Surgical procedures

**Figure-1:**
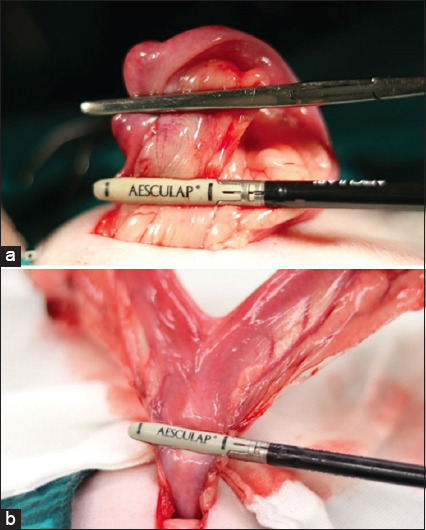
Use of a vessel sealing (VS) device in ovariohysterectomy. (a) VS used in coagulating and cutting the suspensory ligament and the ovarian pedicle. (b) Placement of the VS through the uterine body near the cervix.

The surgical site was routinely clipped and prepared aseptically. Standard ventral midline celiotomy was performed for OHE. The incision line was created from the umbilicus until halfway between the umbilicus and pubis. The procedure began with the identification of the ovary. Hemostatic forceps were placed on the proper ligament of the ovary and used to mark and manipulate the ovary [27]. A window was created in the broad ligament just caudal to the ovarian pedicle. In SL, OHE was performed using the three-clamp technique [27], where both the left and right ovaries were removed after double ligation of the ovarian pedicle and uterine cervix using polyglactin 910 (Kruuse Sacryl, Kruuse, Denmark, USP 3/0–0 according to bodyweight). In VS, both the left and right ovaries were removed after coagulating and cutting the suspensory ligament and ovarian pedicle using a bipolar VS device (Figure-1a) for open surgery (Caiman^®^ Aesculap^®^ Germany, 5 mm blunt jaw, 24 cm in length). The same device was used to coagulate and transect the broad ligament. The proximal cervix was coagulated and cut using the VS device, which was applied approximately 2–3 times across the same location to ensure complete tissue sealing (Figure-1b). The abdomen wall and skin incision were closed using a routine procedure: The linea alba with a simple continuous pattern using polydioxanone (MonoPlus^®^, B. Braun Surgical, Spain); subcutaneous tissues in a subcuticular pattern using glyconate (Monosyn^®^, B. Braun Surgical); and skin in a simple interrupted pattern using polyamide (Dafilon^®^, B. Braun Surgical).

### Surgical time

The surgical duration was divided into three phases as described by Tallant *et al*. [15]. Phase 1 started with a skin incision and ended when one of the ovaries was grasped. Phase 2 started with ovary manipulation and finished with abdominal wall closure. Phase 3 began with the closure of the abdominal wall incision and ended with the suturing of subcutaneous tissue and skin. The total surgical time and any complications were recorded.

### Percentage of blood loss

Based on a protocol described previously [28, 29], gauze was cut into 3 × 3 inches, pre-weighed before use, packed, and sterilized. The weights of the gauze pieces were measured immediately after surgery to avoid errors. The percentage of blood loss during surgery was calculated as the proportion of estimated blood loss volume/79 mL/kg body weight.

### Pain assessment and follow-up

Baseline measurements of pain and physiological data were recorded on the morning of the surgery and before administering any medication perioperatively.

The post-operative pain scores were assessed using the Colorado state university canine acute pain scale (CSU-CAP) [30] and the short form of the Glasgow composite pain scale (SF-GCPS) [15]. Each dog was assessed at 30 min and 1, 2, 3, 4, 24, and 72 h post-operatively. Tramadol hydrochloride (3 mg/kg, IV) was administered for rescue analgesia if dogs obtained scores equal to or greater than the cutoff score for rescue pain on any scale (CSU-CAP ≥2 [31] or SF-GCPS ≥5 [15, 32]).

All dogs were sent home on the same day as the surgery after full recovery from anesthesia, with instructions for post-operative care. All data were recorded by veterinarians who were blinded to the surgical procedure.

## Physiological parameters

Physiological variables, heart rate (beats/min), respiratory rate (breaths/min), systolic blood pressure (mmHg), and body temperature (°F) were recorded during each phase of surgery and post-operatively at the previously specified time points [30, 33].

### Statistical analysis

Data were expressed as the mean and standard error of the mean (SEM). Statistical analyses were separately performed for data obtained at baseline, each surgical phase (phases 1, 2, and 3), and post-operatively (30 min, 1, 2, 3, 4, 24, and 72 h post-operatively). Since the data used were from the same dogs, repeated measures analysis using a mixed model (SAS^®^ University Edition: SAS Institute Inc, Cary, NC.) was performed. Pain scores and physiological parameters, including SF-GCPS, CSU-CAP, respiratory rate, heart rate, systolic blood pressure, and body temperature were the dependent variables. The interactions of the time points and the groups were the independent variables in all models. Autoregression Type 1 was defined as the correlation structure for repeated data. The least squares of means were calculated to define the significant differences in means between the pairwise comparisons. For all comparisons, significance was defined as p < 0.05.

## Results

In total, 28 intact female dogs were included in the study, including mixed breeds (n = 14), Chihuahua (n = 9), Pomeranian (n = 1), Thai Bangkaew (n = 2), Siberian Husky (n = 1), and Shih Tzu (n = 1). The pre-operative overall means and SEM of weight, heart rate, respiratory rate, systolic blood pressure, and body temperature in the SL and the VS groups obtained are shown in Table-1. The mean age was 2.60 and 2.17 years in the SL and VS groups, respectively. No significant differences were found in any of the parameters, indicating the absence of selection bias.

**Table-1:** Overall means and SEM of weight, heart rate, respiratory rate, systolic blood pressure, and body temperature of dogs in the SL and the VS groups obtained before the OHE operation.

Parameter	SL		VS	
	
	Means	SEM	Means	SEM
Weight (kg)	7.1	1.7	10.2	2.5
Heart rate (beats/min)	128.8	6.5	116.5	6.6
Respiratory rate (breaths/min)	33.9	2.9	36.4	5.2
Systolic blood pressure (mmHg)	129.5	6.3	135.1	5.8
Body temperature (^o^F)	101.8	0.2	101.4	0.2

SEM=Standard error of the means, SL=Suture ligation, VS=Vessel-sealing

**Figure-2:**
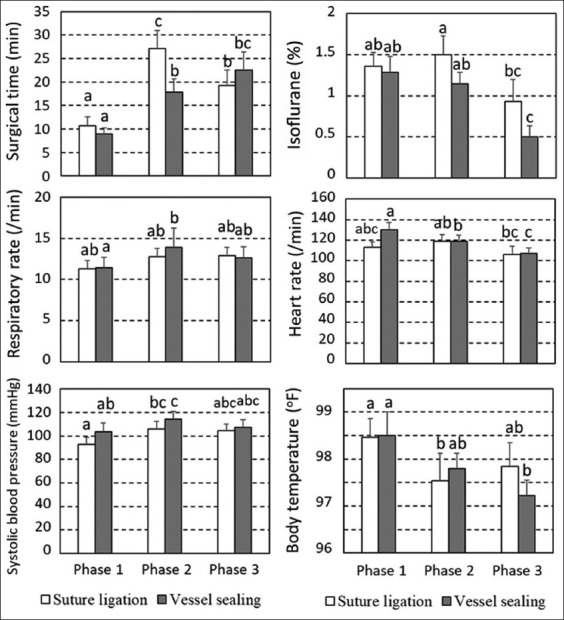
Means and standard error of the means of parameters separated into Phase 1: Skin incision to one of the ovaries being grasped, Phase 2: Ovary manipulation to abdominal wall closure, Phase 3: Closure of the abdominal wall incision to finish suturing the skin. ^a,b,c^different letters indicating significant differences at p < 0.05.

Among 28 dogs, perioperative complications were observed in two dogs, including intraoperative bleeding at the right ovarian pedicle due to the removal of a suspensory ligament with a subsequent performance of an extra-ligation in a dog in the SL group and use of the VS device to stop bleeding and bloody vaginal discharge at 1 h post-operatively in a dog in the VS group, requiring reoperation using extra-ligation at the uterus. The mean duration of the entire surgical procedure was 57.69 ± 27.51 min in the SL group and 43.85 ± 18.95 min in the VS group (p = 0.148). The mean operation times for phases 1, 2, and 3 for SL were 10.7 min (range, 5–30 min), 27.1 min (range, 5–50 min), and 19.3 min (range, 5–45 min), respectively. For VS, the mean operation times for phases 1, 2, and 3 were 8.9 (range, 5–20 min), 17.9 min (range, 5–45 min), and 22.5 min (range, 5–60 min), respectively (Figure-2). The percentage of blood loss did not vary between SL (1.88% ± 2.11%) and VS (0.88% ± 0.60%).

**Figure-3:**
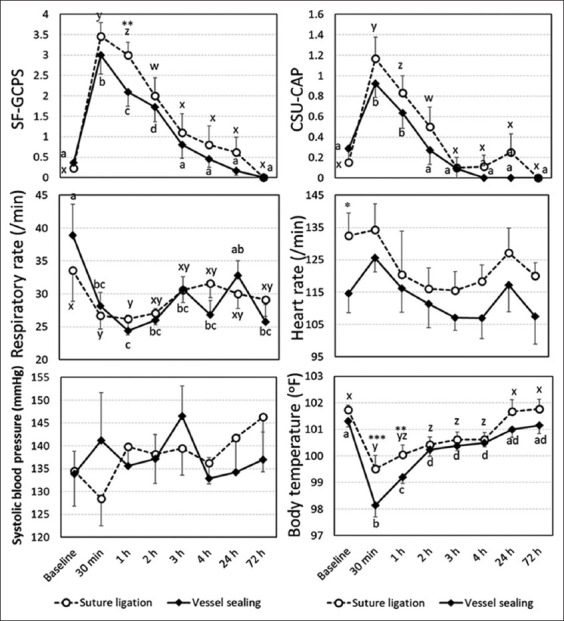
Means and standard error of the means of the pain scores, including SF-GCPS and CSU-CAP, and physiological parameters including heart rate, respiratory rate, systolic blood pressure, and body temperature between SL and VS groups at baseline, 30 min, 1, 2, 3, 4, 24, and 72 h following post-operative care of the SL and VS groups. *, **, *** indicates a significant difference between SL and VS at the specified time following the post-operative care at p < 0.1, p < 0.05, and p < 0.01, respectively. ^w,x,y,z.^ different letters indicate a significant difference at p < 0.05 within the SL group. ^a,b,c,d^different letters indicate a significant difference at p < 0.05 within the VS group. SF-GCPS=Short form of the Glasgow composite pain scale, CSU-CAP=Colorado state university canine acute pain scale, SL=Suture ligation, VS=Vessel sealing.

In phase 2, the surgical duration of SL (27.1 ± 3.77 min) was higher than that of VS (17.9 ± 2.76 min) at p < 0.05, coinciding with the lower isoflurane use (p = 0.20) (Figure-2). Isoflurane use was significantly lower in phase 3 than in phase 2 in both SL and VS. In VS, the respiratory rate in phase 1 (11.4 ± 1.27 breaths/min) was significantly lower than in phase 2 (13.9 ± 2.35 breaths/min). In VS, the heart rate in phase 1 (130.1 ± 7.14 beats/min) was significantly higher than those in phase 2 (118.9 ± 5.81 beats/min) and phase 3 (107.1 ± 5.37 beats/min). Significantly increased systolic blood pressure was observed between phases 1 and 2 in both SL and VS. Body temperature gradually decreased only in VS, and a significant difference was observed between phases 1 and 3.

post-operatively, two dogs in SL each showed signs of serious pain at 30 min and 2 h, respectively. They were subsequently administered with tramadol hydrochloride IV for rescue analgesia. Figure-3 presents the mean ± SEM of the SF-GCPS and CSU-CAP scores and physiological parameters, including heart rate, respiratory rate, systolic blood pressure, and body temperature between SL and VS at baseline; 30 min; and 1, 2, 3, 4, 24, and 72 h following post-operative care. In the comparison between SL and VS at the time points following post-operative care, SF-GCPS at 1 h, heart rate at baseline, and body temperature at 30 min and 1 h of SL were higher than VS at p < 0.05, p < 0.1, p < 0.01, and p < 0.05, respectively. For SF-GCPS, the pain score patterns in both SL and VS were similar, with the peak score was observed at 30 min and gradually decreased with time until it equaled the baseline at 3 h.

In contrast, the CSU-CAP score showed that both SL and VS peaked at 30 min; however, the scores for VS were equal to the baseline level at 2 h, whereas those of SL equaled the baseline at 3 h. The respiratory rates in both SL and VS were lowest at 1 h, which was significantly lower than baseline. The body temperature patterns were similar between SL and VS. The lowest temperatures were at 30 min, and gradually increased to equal those at baseline at 24 and 72 h.

## Discussion

In the present study, OHE performed using a VS device obtained a better early post-operative pain score and had a shorter operating time for the OHE than SL.

Dogs in both groups demonstrated progressive lowering of the pain score during post-operative recovery. We found that SF-GCPS values at 1 h after surgery were significantly lower in the VS group than in the SL group; however, no significant differences were identified at other time points. Moreover, the VS group also showed CSU-CAP values that returned to the baseline level at 2 h post-operatively at a faster rate than that in the SL group. Most likely, using the VS device during OHE can decrease post-operative pain. This is confirmed by a previous study that evaluated post-operative pain using different techniques for OHE; VS resulted in less pain for dogs [5] and less discomfort for cats [18], whereas SL resulted in higher pain scores at 1, 4, and 6 h after surgery [34].

In the SL group, two of the 14 dogs required additional tramadol hydrochloride, whereas no dogs in the VS group required additional pain medication. Interestingly, the SL group showed a significant increase in SF-GCPS post-operatively compared with the VS group, with the greatest increase observed during the clamping of the ovarian pedicle [12] and handling of the suspensory ligament [15]. Höglund *et al*. [35] reported surgical trauma of the suspensory ligament and removal of the ovary during spaying. The SL method required a more caudal retraction of the ovaries to expose the retroperitoneal space [20] and to clamp and ligate the ovarian pedicle, resulting in the rupture of the suspensory ligament [15]. However, less post-operative pain has been associated with the relatively atraumatic process and the sealing and transection of the ovarian pedicle using the VS device [20]. Moreover, ovarian pedicle clamping and ligation produced stimulation of severe pain through the transmission of nociceptive stimuli from the periphery to the central nervous system [12, 36]. We hypothesized that when the suspensory ligament was torn manually and the ovarian pedicle clamped and ligated, the resulting pain was greater than that with the use of a VS device.

Post-operative pain is acute pain associated with tissue damage due to trauma and organ manipulation during OHE [13, 37]. Tissue injury causes the release of inflammatory mediators, including nitric oxide (NO), reactive oxygen species, hydrogen peroxide, and cytokines [38, 39]. Nonsteroidal anti-inflammatory drugs (NSAIDs) are commonly administered to companion animals to relieve pain and reduce inflammation by inhibiting the synthesis of prostaglandin through cyclooxygenase enzyme inhibition [40, 41]. Kılıç and Kozacı [42] investigated the effects of administering metamizole, flunixin meglumine, carprofen, and diclofenac through the intraperitoneal route in rats undergoing laparotomy for 5 days and reported that these drugs can reduce the levels of NO, erythrocyte glutathione, and malondialdehyde, thereby lowering oxidative stress and inflammation. Conversely, administering NSAIDs, such as flunixin, diclofenac, and metamizole, for up to 14 days post-operatively in rats caused delayed wound-healing by reducing wound contraction and angiogenesis at the wound site, which are two important factors in tissue integrity [43]. Thus, NSAIDs should always be used with caution to reduce adverse effects on wound-healing [43]. In our study, all dogs received one dose of carprofen post-operatively, and both groups showed reduced post-operative pain scores. However, inflammatory mediators and the wound-healing process were not evaluated in the present study. Based on the works of Kılıç and Kozacı [42] and Erpek *et al*. [43], we conducted a prospective study on wound-healing and inflammatory mediators after administering NSAIDs.

The total surgical duration in the VS group was shorter than in the SL group, although the difference was not statistically significant. However, the surgical time from the identification of the first ovary to the removal of the uterus was significantly shorter in the VS group than in the SL group. Boursier *et al*. [44] used a bipolar VS device to perform complete OHE in a cat with pyometra and reported that a major reason for the shorter surgical time is that, in using bipolar VS, the ovarian pedicle, broad ligament, and uterine body are sealed and cut.

Physiological changes in heart rate, respiratory rate, and blood pressure occur in animals after noxious stimuli [30, 33]. Heart rate, respiratory rate, and noninvasive blood pressure did not differ significantly between the SL and VS groups perioperatively. A significant change in heart rate and isoflurane use during phase 3 may not be as clinically relevant in other phases of surgery as in this phase since incision closure was similarly performed in both groups. These findings are in agreement with the previous results [15].

A significantly lower respiratory rate compared with the baseline was observed at each time point post-operatively in both groups. Differences in this parameter between the pre- and post-operative periods were not clinically relevant because the pre-operative measurements were most likely influenced by fear, stress, and excitement, which can be triggered in a hospital environment [32, 45] or duringthe first dog–human interaction including white-coat effect and restraint [46]. Furthermore, the comfort of all dogs was ensured by sufficient management of post-operative pain; therefore, they did not demonstrate an increased respiratory rate. Hancock *et al*. [5] described post-operative pain after OHE in dogs and found no significant differences between harmonic scalpel-assisted laparoscopy and median celiotomy with SL in terms of heart rate and respiratory rate. Nevertheless, physiological parameters associated with pain assessment have been used in several studies because they can provide effective information on the response to noxious stimuli [47]. In our opinion, physiological data should be used in conjunction with other measurement techniques to obtain better results; this has also been described in several previous studies [32, 48, 49].

The percentage of blood loss was lower in the VS group compared with the SL group, but the difference was not significant. However, perioperative complications were observed in our study. One dog in the SL group experienced bleeding from the right ovarian pedicle after the release of a suspensory ligament, whereas one dog in the VS group showed bloody vaginal discharge approximately 1 h after surgery. Hemorrhage can cause death [16], and the most common cause of hemorrhage in open OHE is rupture of the ovarian pedicel [17] after insufficient manipulation or knot-tying [25] or during the attempted release of a suspensory ligament [17]. Davidson *et al*. [50] reported that a dog undergoing laparoscopic OHE had bloody vaginal discharge and a blood clot that may have been caused by a parous and large uterine body. This makes sealing more difficult, as the jaw of the sealing device cannot completely pass around the uterine body and securely seal the tissue, thereby delaying uterine VS. We believe that the complications in our study were associated with surgeon experience and a diameter of the uterine body ≥9 mm, which is contraindicated for the sealing device [51]; however, these issues were not observed in our study.

## Limitations of the study

Our study has three main limitations. First, the VS device was applied approximately 2–3 times across the same location at both the ovarian pedicle and uterine body for complete sealing of the soft tissue to prevent technical failure and ensure patient safety. Second, we used a reusable VS device for economic reasons, although it is a single-use medical instrument. Gardeweg *et al*. [21] showed that the same device can be reused up to 5 times without increasing the risk of sealing failure. In our study, the device was reused for five times. Third, we did not record the diameter of the uterine body and the length of the surgical incision in both groups. The extent of soft tissue damage in each group will greatly influence the results of the study. Although the VS device had a higher cost, the results of this study agrees with a previous study of Schwarzkopf *et al*. [25] that this device is more effective in neutering bitches because it is safe, less painful, easy to use, and faster in terms of operation time and can help avoid post-operative complications from suturing materials.

## Conclusion

Our findings reveal that VS in canine OHE via celiotomy can be performed without foreign material remaining in the abdomen. The VS group exhibited significantly less post-operative pain and shorter surgical duration than the SL group, thereby confirming the advantage of this device. Therefore, we can conclude that this device can be used instead of traditional vessel ligation in routine veterinary surgery.

## Authors’ Contributions

JS and PK: Designed the study. JS, PK, and SM: Collected the samples and conducted the study. JS, PK, WAS, WIS, and KW: Supervised the study, analyzed the data, drafting, and editing of the manuscript. All authors have read and approved the final manuscript.
